# Japanese-Language AI Agent System for Human Papillomavirus Vaccine Infoveillance and Public Communication: Development and Feasibility Evaluation

**DOI:** 10.2196/90295

**Published:** 2026-05-21

**Authors:** Junyu Liu, Siwen Yang, Dexiu Ma, Qian Niu, Zequn Zhang, Momoko Nagai-Tanima, Tomoki Aoyama

**Affiliations:** 1 Graduate School of Medicine, Kyoto University, Yoshida-honmachi, Sakyo-ku, Kyoto, 606-8501, Japan, 81 075-753-7531; 2 David R. Cheriton School of Computer Science, University of Waterloo, Waterloo, ON, Canada; 3 Department of Computer Science, Whitacre College of Engineering, Texas Tech University, Lubbock, TX, United States; 4 Graduate School of Engineering, The University of Tokyo, Bunkyo-ku, Tokyo, Japan; 5 Department of EEIS, University of Science and Technology of China, Hefei, Anhui, China

**Keywords:** human papillomavirus, HPV, artificial intelligence agent, AI agent, large language model, stance analysis, topic modeling, artificial intelligence, AI

## Abstract

**Background:**

Human papillomavirus (HPV) vaccine hesitancy remains a significant public health challenge in Japan, where proactive vaccination recommendations were suspended between 2013 and 2021. The resulting information gap between medical institutions and vaccine-hesitant populations is exacerbated by misinformation on social media platforms. Traditional public health communication strategies cannot address individual queries while simultaneously monitoring population-level discourse.

**Objective:**

This study aimed to develop and conduct a feasibility evaluation of a dual-purpose artificial intelligence agent system that delivers verified HPV vaccine information to the public through a conversational interface while generating infoveillance reports for medical institutions based on user interactions and social media discourse.

**Methods:**

We implemented a system with 3 components: a vector database integrating 139,803 documents, including academic papers, Japanese government sources, news media, and social media posts; a retrieval-augmented generation chatbot using a ReAct agent architecture with iterative multitool orchestration across 5 specialized knowledge sources; and an automated report generation system with modules for news analysis, research synthesis, social media sentiment analysis, including stance classification and topic modeling, and user interaction pattern identification. System performance was assessed using both automated and manual evaluation protocols on a scale from 0 to 5.

**Results:**

The entire system functioned as expected. For single-turn evaluation, the chatbot achieved mean scores of 4.83 (SD 0.67; 95% CI 4.71-4.93) for relevance, 4.89 (SD 0.53; 95% CI 4.79-4.97) for routing, 4.50 (SD 1.29; 95% CI 4.27-4.70) for reference quality, 4.90 (SD 0.62; 95% CI 4.78-4.99) for correctness, and 4.88 (SD 0.54; 95% CI 4.78-4.96) for professional identity, with an overall mean of 4.80. Multiturn evaluation yielded higher mean scores: 4.94 for context memory (SD 0.25; 95% CI 4.84-5.00) and an overall mean of 4.98, with topic centering and identity achieving 5.00. The report generation system achieved high scores across all sections: 4.83 for completeness (SD 0.37; 95% CI 4.73-4.94), 4.88 for correctness (SD 0.33; 95% CI 4.77-4.96), and 4.12 for helpfulness (SD 0.48; 95% CI 3.98-4.27). Reference validity achieved perfect scores (5.00) across all periods, with citation correctness averaging 4.21 (SD 0.58; 95% CI 3.96-4.46).

**Conclusions:**

This feasibility study demonstrated that an integrated artificial intelligence agent system can support both public HPV vaccine communication and social media infoveillance in a Japanese-language context. Prospective deployment with real users is needed to assess actual public health impact.

## Introduction

Human papillomavirus (HPV) is a significant public health concern that caused 662,044 new cervical cancer cases and 348,709 deaths worldwide in 2022 [1]. HPV vaccines have demonstrated high efficacy in preventing HPV-related diseases [2], and numerous countries have implemented national vaccination programs since their introduction in 2006. However, vaccine hesitancy remains a persistent challenge [3], particularly in countries such as Japan, where HPV vaccination rates have dropped dramatically owing to safety concerns and media coverage of alleged adverse events [4].

The spread of misinformation regarding HPV vaccines through social media platforms has exacerbated public concern [5], creating a complex information landscape in which accurate medical information competes with anecdotal reports and unverified claims. Traditional public health communication strategies face significant challenges in addressing health misinformation at scale because responding effectively requires simultaneously countering individual-level psychological barriers and monitoring population-level misinformation dynamics across diverse platforms [6]. Medical institutions require timely insights into public discourse to develop effective communication strategies; however, manual analysis of vast amounts of social media data and public inquiries is resource-intensive and time-consuming.

Recent advances in large language model (LLM) and retrieval-augmented generation (RAG) systems offer promising solutions to bridge this information gap [7]. LLMs demonstrate remarkable capabilities for natural language understanding and generation across multiple languages [8], including Japanese, which presents unique challenges owing to its complex writing system and grammatical structure [9]. RAG systems combine the generative capabilities of LLMs with retrieval from curated knowledge bases, enabling responses grounded in verified information sources while maintaining conversational fluency.

Previous studies have applied natural language processing to HPV-related social media analysis, primarily focusing on sentiment analysis and topic modeling [10]. However, these approaches typically operate as passive analytical tools instead of as active information dissemination systems. Chatbot systems for health information have been developed for various domains [11]; however, few integrate multisource retrieval from academic literature, official guidelines, news media, and social media discourse while simultaneously providing bidirectional communication between the public and health institutions.

The Japanese context poses unique challenges to and opportunities for such systems. Japan experienced a dramatic suspension of proactive HPV vaccination recommendations from 2013 to 2021 because of safety concerns, resulting in vaccination rates falling below 1% and creating a substantial gap in population immunity [4]. The government’s 2022 resumption of vaccination recommendations necessitates renewed public education efforts [12]. Furthermore, Japanese-language health information systems face technical challenges, including multiscript processing (hiragana, katakana, and kanji), medical terminology localization, and culturally appropriate communication styles.

In this study, we developed and implemented a comprehensive artificial intelligence (AI) agent system designed to address both public information needs and institutional monitoring requirements for the HPV vaccine discourse in Japan. Our system has two main features: (1) a RAG-based chatbot that answers public queries by retrieving and synthesizing information from academic papers, official documents, news articles, and social media posts; and (2) an analytics dashboard that generates reports for medical institutions based on aggregated chat histories and social media data. The system uses multisource data collection, semantic search with vector embeddings, intelligent query routing, and automated evaluation frameworks.

## Methods

### System Architecture

We developed a multicomponent AI agent system for HPV vaccine information dissemination and public opinion analysis. The system comprises 3 main modules: a multisource data collection and storage system, a ReAct agent–based chatbot [13] for public information queries, and a report generation system for medical institutions.

**Figure 1.**
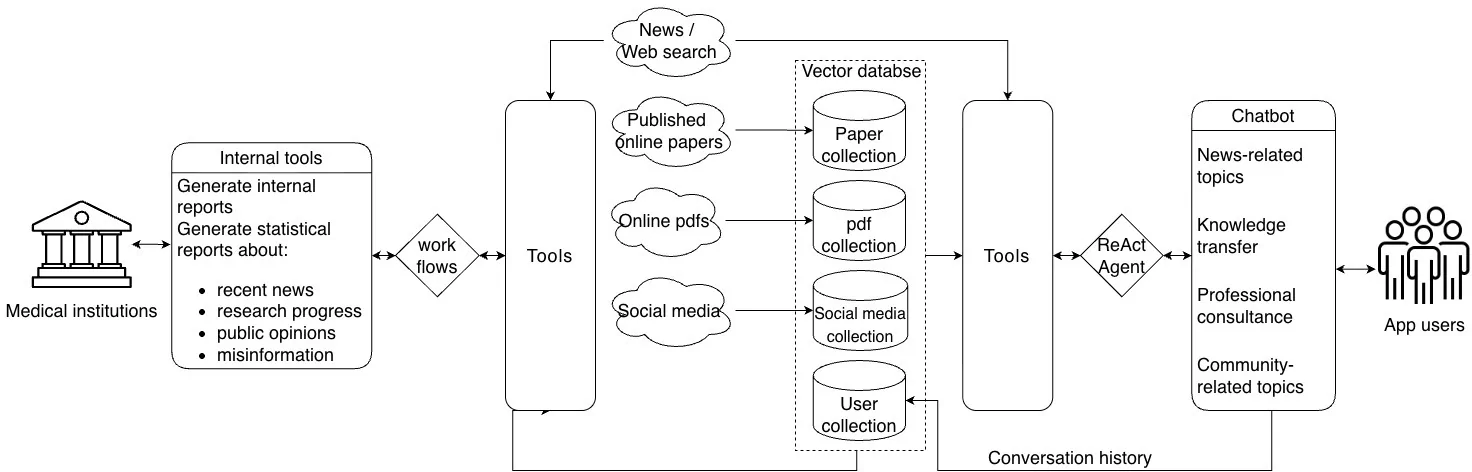
Overall system architecture showing the integration of the data collection, vector database, chatbot interface, and report generation components. HPV: human papillomavirus.

The overall architecture follows a distributed design pattern with a centralized vector database (Qdrant) [14] serving as a knowledge repository (Figure 1). Data flow from multiple external sources through specialized collectors in the database, where they are indexed using semantic embeddings. The chatbot and report generation modules both query this database but serve different end users with distinct interfaces and functionalities. The system implements a bidirectional information flow. The chatbot provides HPV vaccine information to the public while simultaneously collecting user inquiries with consent, and the report generator aggregates these interactions with social media data to produce actionable insights for medical institutions.

### Data Collection and Database

We implemented a vector database infrastructure as the central knowledge repository managing 4 distinct collections: academic papers, official documents, social media posts, and chat histories. Each document was represented as a 2048D vector using embedding models optimized for Japanese-language processing (PLaMo-Embedding-1B; Preferred Networks, Inc) [15]. This database uses cosine similarity metrics for semantic search operations [16,17], supporting efficient retrieval with customizable parameters and metadata preservation.

Data were collected from 4 heterogeneous sources to construct a comprehensive knowledge base spanning scientific evidence, official guidance, media coverage, and public discourse. Academic papers were retrieved from PubMed [18] through keyword-based searches with temporal filtering, capturing abstracts, MeSH (Medical Subject Headings) terms, journal information, and DOIs. Official documents and web content were collected from authoritative sources, including the World Health Organization [19] and the Japanese Ministry of Health, Labor, and Welfare (MHLW) [20] through multiple complementary methods: intelligent query analysis for information synthesis, filtered web searches targeting official sources, online PDF document discovery and extraction, and specialized scraping of government meeting records and reference materials. News articles were aggregated from multiple news sources using keyword-based searches in Japanese and English, and deduplication was used to ensure unique coverage. Social media data from X (formerly known as Twitter) were collected through daily automated harvesting using Tweepy [21] with temporal specifications. Rate limit handling was implemented to ensure comprehensive data capture across extended periods.

### Chatbot Implementation

We implemented a ReAct agent–based chatbot using LlamaIndex [22] through an iterative multitool orchestration architecture in which a single intelligent controller dynamically selects and combines information from multiple specialized data sources across sequential decision-making iterations. The system addresses the challenge of answering diverse user queries by enabling flexible, multisource information gathering while maintaining conversational coherence and citation quality assurance.

#### Architecture

The chatbot uses a single controller agent with 5 specialized tools: papers (academic literature), the web (official documents and guidelines), social media (public discourse), news (media coverage), and chitchat (casual conversations). A citation validation tool ensures the response quality. Unlike conventional routing architectures, this design enables the controller to select and combine multiple tools iteratively for a single query, synthesizing information across heterogeneous sources.

Each tool performs a semantic similarity search against its respective vector database collection, retrieving the relevant documents that the controller assembles into responses using the proper source attribution. The controller analyzes queries in the conversation context, determines appropriate information sources, and iteratively gathers evidence until it is sufficient for comprehensive response synthesis. A web-based Streamlit interface (Snowflake Inc) [23] presents conversations with integrated citations, whereas tool use metadata are stored with user consent to inform institutional reporting.

#### Operational Workflow

**Figure 2.**
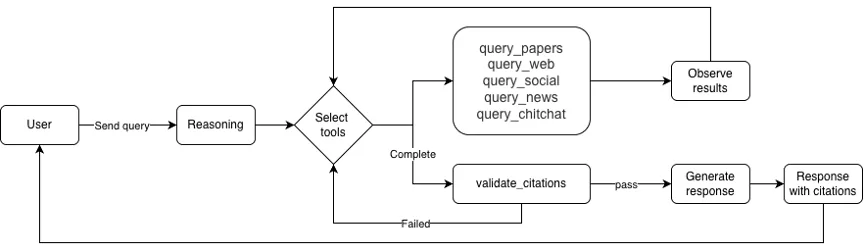
Chatbot operational workflow showing the iterative ReAct agent architecture. The user query flows through reasoning and tool selection, with the controller dynamically selecting from 5 specialized tools (papers, the web, social media, news, and chitchat). Results are observed and validated through a citation validation mechanism before generating the final response with proper source attribution.

The query processing follows an iterative orchestration loop (Figure 2). Upon receiving a user message (query), the controller examines the question along with the recent conversation history to assess the information requirements. The controller then enters a decision cycle: (1) analyze information gaps, (2) select the most appropriate tool, (3) retrieve results via a semantic similarity search, (4) review relevance, and (5) determine whether sufficient evidence exists or additional retrieval is required. This process continues until comprehensive information is gathered for response generation.

The system generates responses using inline citation markers corresponding to the retrieved documents, enabling users to trace claims to their original sources. A 2-level citation validation mechanism ensures quality: individual tools validate their own citations, and a dedicated validation tool examines the entire response for citation completeness before delivery.

Privacy protection is implemented for social media queries, synthesizing themes and sentiment patterns without attributing statements to individual users. Stateful conversation management maintains dialogue context through a windowed history approach, enabling interpretation of follow-up questions with implicit references (eg, “What about side effects?” following a vaccine efficacy discussion) while maintaining topical continuity.

### Report Generation System

We developed an automated report generation system that synthesizes data from multiple sources to produce comprehensive PDF reports for medical institutions and policymakers. This system uses LLMs for intelligent analysis and generates professional documents with academic-style citations, visualizations, and actionable insights.

#### System Architecture

**Figure 3.**
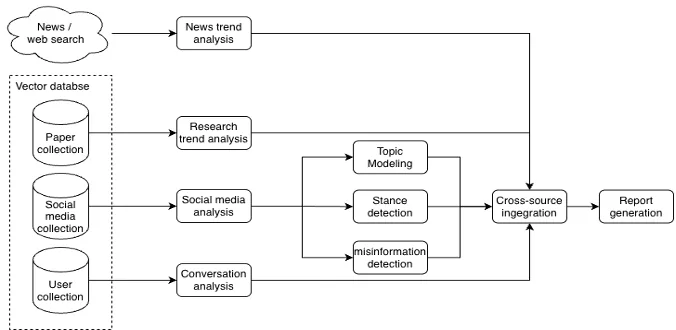
Report generation system architecture. Data flow from external news sources and vector database collections (papers, social media, and user conversations) through specialized analysis modules. The social media analyzer performs topic modeling, stance detection, and misinformation detection. All analysis results are integrated through cross-source aggregation before final report generation.

The report generation system (Figure 3) comprises four specialized analysis modules coordinated by a central orchestrator: (1) news analyzer for recent news, (2) paper analyzer for recent academic research, (3) social media analyzer for public sentiment analysis, and (4) chat analyzer for user interaction pattern identification. Each module queries the vector database for documents within a configurable time window, performs domain-specific analysis using an LLM-based inference, and generates a structured output with properly formatted citations. The orchestrator coordinates module execution, manages data flow between components, aggregates results, and assembles the final PDF document with bilingual support (Japanese and English).

#### Social Media Analysis

Social media platforms have emerged as critical channels for public health discourse, serving as real-time indicators of population-level attitudes toward vaccination [24]. For medical institutions and policymakers, a systematic analysis of social media content provides valuable insights into public concerns, emerging misinformation narratives, and temporal shifts in vaccine sentiment [25].

The social media analyzer performs a multidimensional public opinion assessment through 4 complementary analytical processes. Stance classification categorizes each post as supportive, opposed, or neutral regarding HPV vaccination using batch LLM inference with a temporal context, thereby aggregating daily counts to track sentiment evolution. Topic modeling uses a hybrid approach that combines statistical latent Dirichlet allocation [26] with LLM-based semantic interpretation in which Japanese text undergoes morphological analysis [27], term frequency–inverse document frequency weighting [28], and model training via Gensim (RARE Technologies Ltd) [29] to extract interpretable topic labels from keyword distributions. Misinformation detection uses LLM-based analysis to identify posts containing claims that contradict the established scientific consensus, recording for each flagged post a short statement of the claim for institutional awareness. Visualization generation produces temporal trend graphics and thematic distribution charts that are embedded directly in the reports to enhance interpretability for nontechnical stakeholders.

#### PDF Report Assembly

The report generator produces professional bilingual documents (in Japanese and English) structured into five main sections: (1) news trends, presenting influential media coverage with relevance assessments; (2) research progress, synthesizing recent academic literature; (3) social media analysis, containing sentiment trends, topic distributions, and visualizations; (4) chat analysis, identifying user information needs and knowledge gaps; and (5) overall summary, providing an executive synthesis across all data sources.

Each section includes inline citations with source-appropriate formatting, which enables medical institutions to verify information and assess evidence quality independently. The orchestrator synthesizes the findings from all the analysis modules into an executive summary that provides a comprehensive overview of the reporting period. This multisource integration approach captures diverse perspectives, enabling stakeholders to develop informed HPV vaccination communication strategies and policy interventions.

### Evaluation Framework

We developed a multifaceted evaluation framework comprising complementary assessment protocols for chatbot performance and automated report generation. The framework uses an LLM-based evaluation for scalable assessment and human expert validation for quality assurance.

#### Chatbot Evaluation Methodology

The chatbot evaluation framework assesses system performance through 2 complementary protocols: single-turn evaluation for individual question-answer exchanges and multiturn evaluation for complete conversation quality. Both protocols use LLM-based judges who receive conversation context, tool use information, and scoring rubrics, generating scores on a scale from 0 to 5 (details are provided in Multimedia Appendices 1 and 2) for each dimension, along with written evaluation notes.

To collect test data, 3 volunteers simulated diverse user personas (Multimedia Appendix 3) to create realistic conversations. They posed questions across different personas and topics, and multiturn conversations with the production chatbot system were conducted and stored for subsequent evaluations.

Using the test data, a single-turn evaluation assesses 5 dimensions: relevance, measuring whether the response addressed the question; routing, evaluating the appropriate tool selection for the query type; reference, assessing citation validity and proper source attribution; correctness, verifying factual accuracy against established guidelines; and identity, examining professional medical communication tone. Multiturn evaluation extends these 5 dimensions through 2 additional metrics for conversational coherence: context memory, which assesses the appropriate use of information from previous turns, and topic centering, which evaluates natural conversation flow with logical transitions between related topics.

We randomly selected 20 question-answer pairs for manual scoring by the 3 domain experts to validate the reliability of the automated evaluation. The correlation between the expert and LLM-generated scores was analyzed to assess whether the automated metrics accurately reflected human judgment.

#### Report Generation Evaluation Methodology

Report quality assessment uses 2 complementary evaluation protocols: main text evaluation for content quality and reference evaluation for citation validity. Both protocols were applied by 3 domain experts using the standardized scoring rubrics provided in Multimedia Appendices 4 and 5, assigning scores on a scale from 0 to 5 for each dimension. Each expert scored every report section independently. The main text evaluation assesses 3 dimensions: completeness, measuring structural integrity and whether sections contain well-developed content; correctness, evaluating factual accuracy and proper interpretation of source materials; and helpfulness, examining practical utility and actionable insights for institutional stakeholders. Reference evaluation validates citation quality across 2 dimensions: reference validity, measuring the proportion of cited sources that are accessible and exist in the underlying database, and citation correctness, assessing whether citations properly support the claims made in the report text.

Temporal analysis generates reports for multiple periods to assess system robustness across varying conditions. This approach evaluates both the system consistency and the ability to capture temporal variations in public discourse. Three volunteers read and scored each report independently.

### Ethical Considerations

The knowledge base was constructed exclusively from publicly available data: PubMed abstracts, World Health Organization and Japanese MHLW documents, news articles, and public X posts. Social media data were analyzed only in aggregate; no individual users were identified or quoted.

During chatbot evaluation, 3 volunteer members of the research team generated simulated conversations with informed consent. No personally identifiable information or personal health data were collected. Conversation data and metadata (time stamps and tool use logs) were stored in a secured database accessible only to the research team.

This study was determined to not require institutional review board review in accordance with the Ethical Guidelines for Medical and Biological Research Involving Human Subjects (Ministry of Education, Culture, Sports, Science, and Technology; MHLW; and Ministry of Economy, Trade, and Industry, 2021) [30] because (1) all external data were publicly available and analyzed without individual identification; (2) evaluation participants were consenting research team members, not patients or vulnerable populations; and (3) no clinical interventions or health outcomes were measured.

## Results

### Database

This database integrates 139,803 documents across 4 specialized collections (Table 1). This document volume and distribution enables a realistic simulation of operational use cases.

**Table 1.** Database collection statistics.

Collection	Documents (n=139,803), n (%)	Unique source	Primary language
Papers	51 (0.04)	37 journals	English (100%)
Official documents	6739 (4.82)	MHLW^a^	Japanese (100%)
Social media	132,874 (95.04)	X (formerly known as Twitter)	Japanese (100%)
Chat conversations^b^	139 (0.10)	37 sessions	English (98.6%); Japanese (1.4%)

a MHLW: Ministry of Health, Labor, and Welfare.

b The chat row describes the conversation set used for the chatbot evaluation: 139 turns, each stored as one document, from 37 conversations. Language proportions were computed over the 139 user messages in this set. Primary language for the Papers, Official documents and Social media rows was assigned on the basis of the source of each collection and was not derived from automated language detection.

### Chatbot

On the basis of the large-scale database, we applied simulation of operational use cases.

#### Example Response

Textbox 1 presents a representative question-answer exchange that demonstrates the response characteristics of the chatbot.

Textbox 1. **Example chatbot response demonstrating professional medical tone, structured information delivery, evidence-based recommendations with citations, and personalized guidance.**

**User query**

“Are there any specific HPV-related health risks or symptoms that become more common or concerning for people over 65?”

**Chatbot response**

“Thank you for asking such an important question about HPV-related health risks for people over 65. As a healthcare provider, I want to address your concerns with both the latest medical evidence and an understanding of the unique considerations for older adults.

*Key Health Risks and Symptoms After 65*

While HPV infections can occur at any age, there are some important patterns to

understand for people over 65. Research shows that while new HPV infections become less common with age, when cervical abnormalities do occur in older adults, they tend to be more significant [1].

...

*References:*

[1] Prevalence of cervical cytology abnormalities and its correlation with human papillomavirus infection. Medicinski glasnik (2025). DOI: 10.17392/1968-22-02”

#### Evaluation Results

The chatbot system was evaluated using simulated conversations generated by 3 volunteers. Conversations spanned diverse user personas of varying age groups, occupations, and levels of concern about HPV vaccination. The questions covered multiple information domains, including vaccine safety, efficacy, eligibility criteria, and procedural guidelines. Table 2 summarizes the single-turn evaluation results (Multimedia Appendix 6) for all 5 assessment dimensions. Average scores ranged from 4.50 to 4.90 on the scale from 0 to 5, with correctness (4.90, 95% CI 4.78-4.99) and routing (4.89, 95% CI 4.79-4.97) achieving the highest scores. Across all dimensions, 90% (125/139) to 99% (137/139) of responses received scores of 4 or higher.

**Table 2.** Single-turn evaluation results (n=139 question-answer pairs).

Dimension	Score (0-5), mean (SD; 95% CI)^a^	Score (0-5), median (IQR)	Score of 5, %	Score of ≥4, %	Score of ≤3, %
Relevance	4.83 (4.71-4.93)	5.00 (5.00-5.00)	91	96	4
Routing	4.89 (4.79-4.97)	5.00 (5.00-5.00)	94	97	3
Reference	4.50 (4.27-4.70)	5.00 (5.00-5.00)	81	90	10
Correctness	4.90 (4.78-4.99)	5.00 (5.00-5.00)	96	99	1
Identity	4.88 (4.78-4.96)	5.00 (5.00-5.00)	93	99	1

a Overall mean 4.80.

A comparison of multiturn with single-turn evaluations revealed consistent improvements (Table 3; details are provided in Multimedia Appendix 7); the overall average increased from 4.80 to 4.98 (+0.18). Topic centering and identity both achieved perfect scores of 5.00 in multiturn settings, indicating that the chatbot maintained natural conversation flow and a consistent professional tone across extended dialogues. Across all dimensions, all responses received scores of 4 or higher.

**Table 3.** Multiturn evaluation results (n=31 conversations).

Dimension	Score (0-5), mean (SD; 95% CI)^a^	Score (0-5), median (IQR)	Score of 5, %	Score of ≥4, %	Score of ≤3, %
Context memory	4.94 (4.84-5.00)	5.00 (5.00-5.00)	94	100	0
Topic centering	5.00 (5.00-5.00)	5.00 (5.00-5.00)	100	100	0
Identity	5.00 (5.00-5.00)	5.00 (5.00-5.00)	100	100	0

a Overall mean 4.98.

We compared the automated scores with human expert assessments to validate the reliability of the LLM-based evaluation. Three domain experts independently scored randomly selected subsets of conversations (n=20 question-answer pairs for single-turn evaluation and n=8 conversations for multiturn evaluation). We report 3 complementary agreement metrics: mean absolute difference (MAD) between averaged expert and LLM scores, Spearman rank correlation coefficient (ρ) for item-level ranking agreement, and intraclass correlation coefficient (ICC(3,1)) for interrater reliability among the 3 experts. Table 4 lists the validation results.

**Table 4.** Agreement with human experts and interrater reliability.

Check type and dimension	Rater 1	Rater 2	Rater 3	MAD^a^	Spearman ρ (95% CI)	ICC^b^(3,1) (95% CI)
Single-turn evaluation (n=20 conversations)						
Relevance	0	0.10	0.05	0.05	0.609 (0.227 to 0.828)^c^	0.203 (−0.057 to 0.511)
Routing	0.40	1.25	0	0.55	—^d^	0.279 (0.008 to 0.575)
Reference	0.60	0.75	0.05	0.47	0.068 (−0.386 to 0.496)	0.170 (−0.085 to 0.481)
Correctness	0.05	0.20	0	0.08	—	−0.025 (−0.229 to 0.281)
Identity	0.15	0.05	0.55	0.25	—	0.314 (0.040 to 0.603)
Multiturn evaluation (n=8 conversations)						
Context memory	0.13	0.13	0.13	0.13	—	—^e^
Topic centering	0	0.13	0	0.04	—	0.000 (−0.307 to 0.545)
Identity	0	0.13	0.50	0.21	—	0.174 (−0.208 to 0.684)

a MAD: mean absolute difference. Overall MAD: 0.28 for single-turn evaluation and 0.13 for multiturn evaluation.

b ICC: intraclass correlation coefficient.

c *P*<.01.

d The large language model scores were all the same, and correlation could not be calculated.

e The 3 experts’ scores were all the same, and the ICC could not be calculated.

For single-turn evaluation, relevance (MAD=0.05) and correctness (MAD=0.08) exhibited close LLM-human alignment, whereas routing (MAD=0.55) and reference (MAD=0.47) showed larger deviations. The overall MAD of 0.28 on a scale from 0 to 5 represents a deviation of less than 6% from human judgment. The Spearman ρ was computable only for relevance (ρ=0.609, 95% CI 0.227-0.828; *P*=.004) and reference (ρ=0.068; *P*=.78) as the LLM assigned uniform perfect scores for the remaining dimensions, leaving no variance for correlation analysis. This ceiling tendency of the LLM judge is itself a notable finding. Interrater reliability among the 3 experts was low to moderate (ICC range −0.025 to 0.314), indicating limited consensus even among human raters, particularly for correctness (ICC=–0.025) and reference (ICC=0.170). Per-rater analysis revealed that individual LLM-expert disagreement varied substantially (eg, routing MAD ranged from 0.00 to 1.25 across raters), suggesting that the observed LLM-human discrepancies partly reflect genuine interexpert disagreement rather than systematic LLM bias.

The multiturn evaluation exhibited closer overall alignment (MAD=0.13; 2.6% deviation), with topic centering achieving near-perfect agreement (MAD=0.04). All 3 experts assigned identical scores to every conversation for context memory, whereas topic centering (ICC=0.000) and identity (ICC=0.174) showed that the small deviations among raters did not follow consistent patterns—a consequence of near-ceiling score distributions. These results suggest that LLM-based evaluation serves as a reasonable proxy for human judgment on dimensions with clear-cut criteria (relevance and correctness) but should be interpreted cautiously for dimensions involving subjective judgment (routing and reference quality), where both the LLM and human raters exhibited greater variability.

### Report Generation

The report generation system was evaluated for 4 distinct periods: January 2020, July 2020, September 2020, and October 2020. We provide an example report in Multimedia Appendix 8. For each period, the system generated complete reports by analyzing 30 days of data from all source collections. Three evaluators independently scored each report section, and we reported the mean with 95% bootstrap CIs across evaluators. Both main text assessment (completeness, correctness, and helpfulness) and reference validation (reference validity and citation correctness) protocols were applied to each report section. The detailed scoring results are presented in Multimedia Appendix 9.

Table 5 summarizes the main text and reference evaluation results. Completeness and correctness both exhibited strong ceiling effects, with medians of 5.00 (IQR 5.00-5.00) and 100% (48/48) of responses achieving scores of 4 or above. Helpfulness showed lower performance (median 4.00, IQR 4.00-4.00; mean 4.12, 95% CI 3.98-4.27), with only 19% (9/48) of responses achieving the maximum score, suggesting room for improvement in generating actionable insights. Reference validity achieved perfect scores across all evaluations (mean 5.00, 95% CI 5.00-5.00). Citation correctness was the most variable dimension (median 4.00, IQR 4.00‐5.00; mean 4.21, 95% CI 3.96‐4.46), with scores spanning the range of 3 to 5 and only 29% (7/24) of responses achieving perfect scores, indicating that the accuracy of citation-to-claim matching is the primary area for improvement in the report generation pipeline.

**Table 5.** Report main text and reference evaluation results.

Dimension	Score (0-5), mean (SD; 95% CI)	Score (0-5), median (IQR)	Score of 5, %	Score of ≥4, %	Score of ≤3, %
Completeness	4.83 (4.73-4.94)	5.00 (5.00-5.00)	83	100	0
Correctness	4.88 (4.77-4.96)	5.00 (5.00-5.00)	88	100	0
Helpfulness	4.12 (3.98-4.27)	4.00 (4.00-4.00)	19	94	6
Reference validity	5.00 (5.00-5.00)	5.00 (5.00-5.00)	100	100	0
Citation correctness	4.21 (3.96-4.46)	4.00 (4.00-5.00)	29	92	8

## Discussion

### Principal Findings

This study demonstrated the feasibility of a dual-purpose AI agent system for HPV vaccine information communication in Japan. This system integrates heterogeneous data sources (academic literature, government documents, news media, and social media) into a unified retrieval infrastructure that supports public-facing conversational interfaces and institutional analytical reporting.

In this preliminary evaluation, the chatbot achieved single-turn medians of 5.00 across all 5 dimensions (IQR 5.00-5.00), with means ranging from 4.50 (reference) to 4.90 (correctness). The pronounced ceiling effects indicate generally high performance but limit fine-grained differentiation between dimensions. Top-box analysis revealed that reference quality received lower scores (14/139, 10% at or below a score of 3 compared to a range of 2/139, 1% to 6/139, 4% for other dimensions), identifying citation handling as the primary area for improvement. Multiturn evaluation showed even stronger ceiling effects (overall mean 4.98), with topic centering and identity both achieving perfect scores across all 31 conversations. The overall mean improvement from single-turn to multiturn evaluation (+0.18) should be interpreted cautiously as it primarily reflects the elimination of low-scoring outliers rather than a broad performance shift. These preliminary results suggest that the iterative multitool orchestration architecture may maintain factual accuracy while delivering appropriately toned medical communication, although validation with real users is needed.

The report generation system maintained consistent quality across 4 temporal evaluation periods. Completeness and correctness exhibited strong ceiling effects (median 5.00, IQR 5.00-5.00), confirming reliable document structure and factual accuracy regardless of data availability fluctuations. Helpfulness was the most variable main text dimension (median 4.00, IQR 4.00-4.00; 9/48, 19% of responses achieving perfect scores), suggesting that, while the system reliably produces structurally complete and accurate reports, generating actionable institutional insights—particularly for chat analysis sections—remains a challenge. Citation correctness showed the widest spread among all report dimensions (median 4.00, IQR 4.00-5.00; 7/24, 29% of responses achieving perfect scores), indicating that the system consistently identifies valid sources (reference validity of median 5.00, IQR 5.00-5.00 across all periods) but is less consistent in matching citations to the specific claims they support. This distinction highlights an important design consideration for automated reporting systems: source retrieval and citation-claim alignment require different optimization strategies.

### Interpretation

These findings suggest that LLM-based RAG systems may address the information asymmetry between medical institutions and vaccine-hesitant populations. The chatbot architecture differs from traditional static frequently asked question systems in that it dynamically selects and combines information from specialized knowledge sources, enabling responses that integrate academic evidence with official guidelines and contemporary public discourse.

The observed improvements in the multiturn evaluation merit consideration. The high context memory scores suggest that the controller incorporates information from previous turns, such as retaining user demographic information when providing age-specific recommendations. Topic centering scores suggest smooth transitions between related topics, resembling the natural progression of clinical consultations from symptoms to screening and prevention. These patterns indicate that the windowed conversation history approach provides sufficient context for a coherent extended dialogue. The low interrater reliability (Table 4) among human experts in single-turn evaluation (ICC range −0.025 to 0.314) may partly reflect the inherent ambiguity of judging individual question-answer pairs in isolation, where raters lack conversational context to disambiguate routing and reference quality assessments.

A 2-level citation validation mechanism may contribute to maintaining response quality. Reference scores confirm consistent source attribution, addressing a key concern in health information systems where users must verify claims independently. Although prospective studies are required to confirm this relationship, transparency may also contribute to user trust.

For institutional stakeholders, the report generation system offers capabilities that would otherwise require substantial manual effort. The consistent structural completeness across the evaluation periods and sections demonstrated reliable document generation regardless of data availability fluctuations. Per-section analysis (Multimedia Appendix 10) reveals that paper sections consistently achieved the highest scores, whereas chat sections exhibited more variability in helpfulness. The September 2020 social media section maintained acceptable quality despite sparser data, suggesting robustness to temporal variation.

Japanese-language implementation addresses challenges specific to this context: multiscript processing, medical terminology localization, and culturally appropriate formal communication. The successful integration of Japanese government documents with English-language research literature provides initial evidence of the viability of this approach in settings where scientific evidence and public health communication occur in different languages.

### Comparison With Prior Work

This work extends previous HPV vaccine natural language processing research, which has primarily focused on passive social media analysis [10], by implementing a bidirectional information flow. Prior health chatbots have been typically retrieved from single knowledge sources [11], whereas our iterative multitool architecture integrates 4 heterogeneous collections, enabling responses that synthesize information across source types.

The evaluation framework extends beyond typical single-turn RAG assessments [31,32] by incorporating a multiturn conversation analysis with simulated users. This approach captures the dimensions of conversational coherence, context memory, and topic continuity that single exchange evaluations overlook.

### Limitations

This study has several limitations that warrant consideration. Most fundamentally, this work represents a proof-of-concept system evaluation rather than a clinical or public health impact study. The system was not tested with real patients, members of the public, or clinical end users in an operational setting. The evaluation relied on simulated conversations generated by 3 volunteers and report assessments by 3 evaluators over 4 periods, which can be insufficient to support strong generalizable conclusions. Additionally, the social media data were exclusively derived from Japanese X users, potentially underrepresenting the older adult population and those with limited digital access. In addition, the LLM-based evaluation may introduce biases that differ from human judgment, particularly for nuanced routing decisions in which multiple valid tool selections exist. The evaluation datasets (139 single-turn exchanges and 31 multiturn conversations) may not capture all real-world interaction patterns, and keyword-based data collection introduces potential selection bias. Geographic specificity to the Japanese government constrains transferability to national contexts with different regulatory frameworks and vaccination policies.

### Future Directions

Several directions warrant further investigation. Future prospective studies with real users and measurable health outcomes are needed to assess the system’s impact on vaccine knowledge, trust, and decision-making, whereas A/B testing will assess whether citation transparency affects user trust. Expansion to other languages and comparative studies across vaccine types would test the generalizability and inform the design principles for vaccine information communication systems facing similar challenges in other contexts.

### Conclusions

This study demonstrated the initial feasibility of an AI agent system that simultaneously addresses public HPV vaccine information needs and institutional discourse monitoring in Japan. The integrated architecture may enable bidirectional information flow—providing verified information with transparent source attribution to users while generating analytical reports for institutional stakeholders—creating links between public concerns and communication strategies. Although this evaluation relied on simulated users, this study established a proof of concept for an AI-augmented vaccine information communication infrastructure, with the transferable architecture and evaluation frameworks providing foundations for adaptation to other vaccines and health conditions and multilingual public health contexts.

## Supplementary material

10.2196/90295 Multimedia Appendix 1 Single-turn chat scoring metrics.

10.2196/90295 Multimedia Appendix 2 Multiple-turn chat scoring metrics.

10.2196/90295 Multimedia Appendix 3 Guidance for the volunteers to chat with the chatbot with different personas.

10.2196/90295 Multimedia Appendix 4 Main text scoring metrics for the generated report.

10.2196/90295 Multimedia Appendix 5 Reference scoring metrics for the generated report.

10.2196/90295 Multimedia Appendix 6 Chat single-turn evaluation results.

10.2196/90295 Multimedia Appendix 7 Chat multiple-turn evaluation results.

10.2196/90295 Multimedia Appendix 8 Example of a generated report.

10.2196/90295 Multimedia Appendix 9 Scores for the generated reports.

10.2196/90295 Multimedia Appendix 10 Section-level evaluation results for the generated reports.

10.2196/90295 Multimedia Appendix 11 Record of chat with artificial intelligence tool.

## Abbreviations

AI: artificial intelligence
HPV: human papillomavirus
ICC: intraclass correlation coefficient
LLM: large language model
MAD: mean absolute difference
MeSH: Medical Subject Headings
MHLW: Ministry of Health, Labor, and Welfare
RAG: retrieval-augmented generation
