# Supplementary material for: Japanese-Language AI Agent System for Human Papillomavirus Vaccine Infoveillance and Public Communication: Development and Feasibility Evaluation
Source: JMIR Infodemiology. 2026 May 21;6:e90295. doi: 10.2196/90295 (PMC13193703; doi:10.2196/90295)
Supplement: Multimedia Appendix 3 [file infodemiology-v6-e90295-s003.docx]

Chat persona simulation guidance

We will randomly choose one item from each of the section, and let the volunteer to do the conversation under this persona.

GENDERS:

- male
- female
- non-binary

AGE_RANGES:

- 18-25
- 26-35
- 36-45
- 46-55
- 56-65
- 65+

OCCUPATIONS:

- Student
- office worker
- healthcare worker
- teacher
- parent
- researcher

LANGUAGES:

- English
- Japanese
