# Supplementary material for: Japanese-Language AI Agent System for Human Papillomavirus Vaccine Infoveillance and Public Communication: Development and Feasibility Evaluation
Source: JMIR Infodemiology. 2026 May 21;6:e90295. doi: 10.2196/90295 (PMC13193703; doi:10.2196/90295)
Supplement: Multimedia Appendix 8 [file infodemiology-v6-e90295-s008.pdf]

HPV Vaccine Monthly Report

Comprehensive Analysis of Research, Media, and Public Discourse in the recent one month

Report Date: 2020-10-31

HPV Vaccine Evaluation Study

1. News Trends

Recent developments in human papillomavirus (HPV) vaccination highlight both significant challenges and promising advancements in cervical cancer prevention. A critical concern is the marked decline in HPV vaccination rates observed in Japan, which has been projected to result in a substantial increase in cervical cancer incidence and mortality. Research conducted by Osaka University estimates that this reduction in vaccine uptake could lead to a rise in avoidable cancer-related morbidity and deaths, underscoring the urgent need for effective public health interventions to reverse this trend and improve vaccination coverage [1].

In contrast to these challenges, advancements in vaccine technology offer considerable potential to enhance preventive efforts. The introduction of the 9-valent HPV vaccine represents a major step forward, as it targets nine HPV types responsible for approximately 90% of cervical cancer cases. This expanded coverage surpasses that of earlier vaccines, which targeted fewer HPV strains, and is expected to significantly reduce the burden of cervical cancer when integrated into vaccination programs. The broader protection afforded by the 9-valent vaccine may also contribute to lowering healthcare costs associated with cancer treatment, thereby improving overall public health outcomes [2].

Further reinforcing the importance of HPV vaccination, recent reports have confirmed the vaccine’s efficacy in preventing HPV-related cancers, particularly cervical cancer. Increased awareness of the vaccine’s cancer-preventive effects is critical for shaping public health policies and encouraging higher vaccination rates. Enhanced understanding among healthcare providers and the public can facilitate informed decision-making and support strategies aimed at reducing the incidence of HPV-associated malignancies [3].

Collectively, these developments emphasize the dual necessity of addressing declining vaccination rates while leveraging improved vaccine formulations to optimize cervical cancer prevention. Coordinated efforts in public health education, policy implementation, and vaccine accessibility remain essential to mitigate the projected rise in cervical cancer cases and to capitalize on the protective benefits of the latest HPV vaccines.

References

[1] HPV ワクチン接種率の激減によって増加する子宮頸がん罹患・死亡者の推計人数 - 大阪大学. 大阪大学, 2020-10-21T07:00:00Z. https://news.google.com/rss/articles/CBMiZkFVX3lxTE9seXV2V0xQWGxjUFZLMFc1d3p1R3Y5SFJfd1ZieS1TeHlVRXg3eWI1MnR3RzFQbVpjUTcyVlcyS3hTRXZXNmQ5TDJMZGFLQ3FleFJnaS1JMHp1YlJOXzBrVkNieHZodw?oc=5

[2] 9価HPVワクチン、子宮頸癌の原因となるHPV型の9割に対応 - 日経メディカル. 日経メディカル, 2020-10-23T07:00:00Z. https://news.google.com/rss/articles/CBMigwFBVV95cUxQNVQzT0o4RnhSUm5FVk5jLUNxX0EzVUxNVzVnbHFMTFVvOUh2ajRKdkx3UzNLSXR2LXBOTk1ua2pWc1FPWUNYNTB0ci1xT3FYN3JvWkxiejJ3UjNMVDFzVWI2Y3g2djRrT2x0empiWEJsS3NUMlI2bkN6Vzk5cVZtUDVxMA?oc=5

[3] HPVワクチン がん予防効果判明と接種の考え方 - 毎日新聞. 毎日新聞, 2020-10-21T07:00:00Z. https://news.google.com/rss/articles/CBMifEFVX3lxTE81TkV1aGlNSlptRmdpZ1FPQjBXdEQ1U2J1MHVwaXd2OGRVT01vZkstVDhvU0JHcHN4WHRRR05pMHJoc0lCTDVmSmlLTnNSYl9PUElWMjI4SS1ERVBKQTIzRj

hUZzBVM2Q1b3c1bVhsc0o2LVYxb2JKcXY4RnI?oc=5

2. Advanced Research Progress

Recent advances in human papillomavirus (HPV) research have concentrated on improving vaccine uptake, expanding therapeutic applications, refining immunological assessment methods, and optimizing cost-effectiveness strategies to enhance public health outcomes globally. A prominent theme is the targeted promotion of HPV vaccination among underserved or high-risk populations, such as LGBTQ individuals, where dermatologists are identified as critical advocates and vaccinators to address existing coverage gaps and reduce HPV-associated morbidity in these communities [1]. Similarly, understanding healthcare providers' perspectives, as exemplified by studies in China, reveals that mixed knowledge levels and cultural sensitivities pose significant barriers to vaccine acceptance, underscoring the necessity for tailored educational interventions and policy reforms to increase vaccination rates in diverse sociocultural contexts [2].

Technological innovations have also emerged as pivotal tools in combating vaccine hesitancy and improving immunization rates. The development of mobile applications like Vaccipack demonstrates the potential of digital health solutions to enhance parental knowledge and intention to vaccinate adolescents, thereby addressing behavioral barriers and facilitating cervical cancer prevention efforts [3]. Complementing these efforts, research highlights the importance of leveraging routine healthcare encounters—including dental and medical visits—as strategic opportunities to promote HPV vaccination, given the observed association between regular healthcare exposure and increased vaccine uptake [5]. These findings collectively emphasize a multidisciplinary and multi-setting approach to vaccination advocacy, integrating clinical practice with public health outreach.

Beyond preventive vaccination, recent studies have explored novel therapeutic applications of the HPV vaccine. Notably, the quadrivalent HPV vaccine has shown promising efficacy in treating recalcitrant non-genital warts resistant to conventional therapies, suggesting an expanded clinical role for the vaccine beyond its traditional use in preventing genital HPV infections and related cancers [4]. This therapeutic potential may influence future clinical guidelines and broaden the scope of HPV vaccine utilization.

Advancements in immunological assessment techniques have further refined the evaluation of vaccine-induced immunity. The introduction of a pseudovirion-based ELISA method offers a sensitive, high-throughput approach to detect HPV-specific antibodies across multiple subtypes, improving upon traditional serological assays. This methodological breakthrough enhances the capacity to monitor immune responses post-vaccination and infection, thereby supporting more accurate assessments of vaccine efficacy and population-level immunity [6].

From a public health and economic perspective, research into the cost-effectiveness of HPV vaccine implementation reveals significant variability in country-level investment

decisions, reflecting diverse willingness-to-pay thresholds. These findings advocate forcontext-specific cost-effectiveness analyses rather than uniform global standards,facilitating more efficient allocation of resources tailored to local economic conditions [7].However, scaling up immunization programs, particularly in low- and middle-incomecountries, remains financially challenging, with substantial investments required to sustainvaccine delivery and achieve broad coverage. Strategic health financing and internationalsupport are therefore critical to overcoming these economic barriers and reducing theglobal burden of HPV-related diseases [9].

Finally, addressing vaccine hesitancy remains a persistent challenge, as evidenced by family physicians’ reports of parental refusal driven by misinformation and safety concerns. These insights highlight the urgent need for targeted communication strategies and provider training to counteract hesitancy and improve vaccine acceptance, which is essential for maximizing the public health impact of HPV vaccination programs [8].

In summary, the current trajectory of HPV research integrates clinical innovation, behavioral science, immunological advancements, and economic evaluation to enhance vaccine uptake and therapeutic applications. These multidisciplinary efforts collectively aim to reduce HPV-associated morbidity and mortality through improved vaccination strategies, expanded clinical indications, and optimized resource utilization across diverse populations and healthcare settings.

References

[1] . Human papillomavirus vaccination in lgbtq patients: the need for dermatologists on the front lines.. Cutis, 2020. DOI: 10.12788/cutis.0084

[2] . Chinese Vaccine Providers' Perspectives on the HPV Vaccine.. Global pediatric health, 2020. DOI: 10.1016/j.canep.2012.01.009

[3] . Vaccipack, A Mobile App to Promote Human Papillomavirus Vaccine Uptake Among Adolescents Aged 11 to 14 Years: Development and Usability Study.. JMIR nursing, 2020. DOI: 10.1542/peds.2016-4186

[4] . The Quadrivalent Human Papillomavirus Vaccine in Recalcitrant Non-genital Warts: A Retrospective Study.. Annals of the Academy of Medicine, Singapore, 2020. DOI: No DOI available

[5] . Association of human papillomavirus vaccination with exposure to dental or medical visits.. Journal of public health dentistry, 2020. DOI: 10.1111/jphd.12408

[6] . Measurement of Human Papillomavirus-Specific Antibodies Using a Pseudovirion-Based ELISA Method.. Frontiers in immunology, 2020. DOI: 10.1016/j.vaccine.2011.07.007

[7] . Informing Global Cost-Effectiveness Thresholds Using Country Investment Decisions: Human Papillomavirus Vaccine Introductions in 2006-2018.. Value in health : the journal of the International Society for Pharmacoeconomics and Outcomes Research, 2020. DOI: 10.1016/j.jval.2020.07.012

[8] . Kansas Family Physicians Perceptions of Parental Vaccination Hesitancy.. Kansas journal of medicine, 2020. DOI: 10.17161/kjm.vol13.14761

[9] . Costs of Immunization Programs for 10 Vaccines in 94 Low- and Middle-Income Countries From 2011 to 2030.. Value in health : the journal of the International Society for Pharmacoeconomics and Outcomes Research, 2020. DOI: 10.1016/j.vaccine.2004.02.029

3. Social Media Analysis

Overall Sentiment and Stance

Analysis of 5,378 tweets related to HPV vaccines reveals a predominantly supportive sentiment on social media platforms. Specifically, 58.2% of the tweets expressed a positive stance towards HPV vaccination, indicating broad public endorsement. Neutral tweets accounted for 28.4%, reflecting a substantial portion of the discourse that is informational or non-committal. Conversely, oppositive tweets comprised 13.4%, representing a minority but notable segment of skepticism or resistance.

The daily stance distribution curve (Figure 1) illustrates fluctuations in public opinion over time, with supportive tweets consistently outnumbering oppositive ones. This trend suggests sustained advocacy and acceptance of HPV vaccination within the online community. The presence of neutral content further indicates ongoing engagement and information-seeking behavior among users, which may serve as an opportunity for targeted educational interventions.

Main Discussion Topics

The thematic analysis identified three primary topics dominating the social media conversation on HPV vaccines. The most prevalent topic, accounting for 42.4% of tweets, centers on HPV vaccination and gender. Discussions frequently address vaccination effects in both males and females, incorporating medical advice and vaccination schedules. This focus underscores the importance of gender-inclusive vaccination strategies and reflects public interest in clinical guidance.

The second major topic, comprising 22.4% of tweets, involves the media portrayal of HPV vaccination, notably referencing the Japanese drama 'コウノドリ.' This series is recognized for its realistic depiction of HPV vaccination and cervical cancer, highlighting the role of popular media in shaping public perceptions and awareness. The third topic, representing 6.4% of tweets, encompasses concerns and discussions about HPV risks, including safety issues raised by pediatricians and broader social implications. This segment reflects ongoing public apprehension and the need for clear communication from healthcare professionals.

Figure 2, the topic distribution bar plot, visually summarizes these thematic proportions, emphasizing the dominance of gender-related vaccination discourse and the significant influence of media representation.

Misinformation Analysis

Misinformation was identified in 655 tweets, constituting 12.2% of the total sample. The false claims predominantly pertain to vaccine safety, efficacy, and alleged adverse effects, which contribute to public uncertainty and vaccine hesitancy. Such misinformation poses a challenge to public health efforts by potentially undermining confidence in HPV vaccination programs.

The presence of misinformation highlights the critical need for proactive and accurate health communication strategies. Addressing these false narratives through evidence-based messaging and engagement with trusted healthcare providers is essential to mitigate their impact. Furthermore, monitoring social media platforms for emerging misinformation trends can inform timely interventions to preserve public trust and promote vaccine uptake.

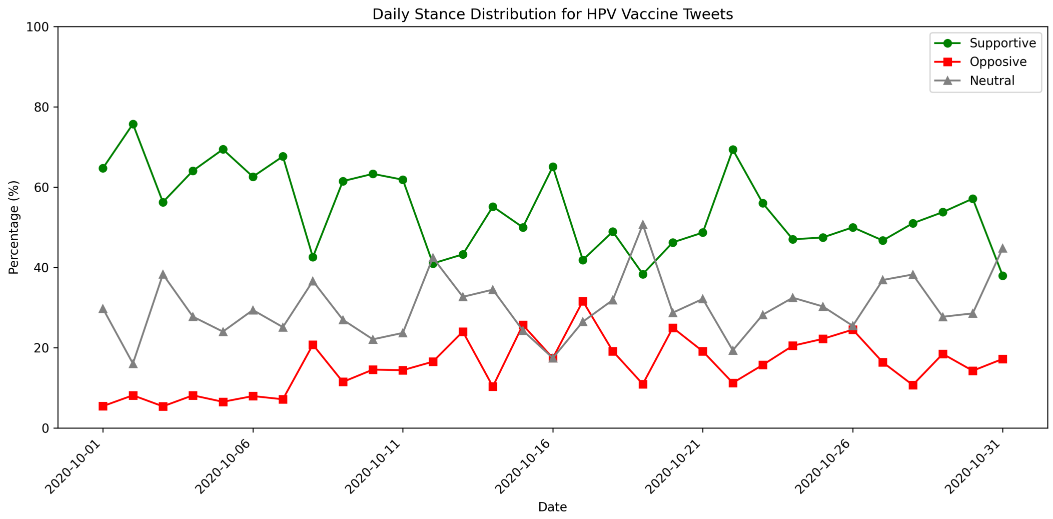

Figure 1: Daily stance distribution for HPV vaccine tweets

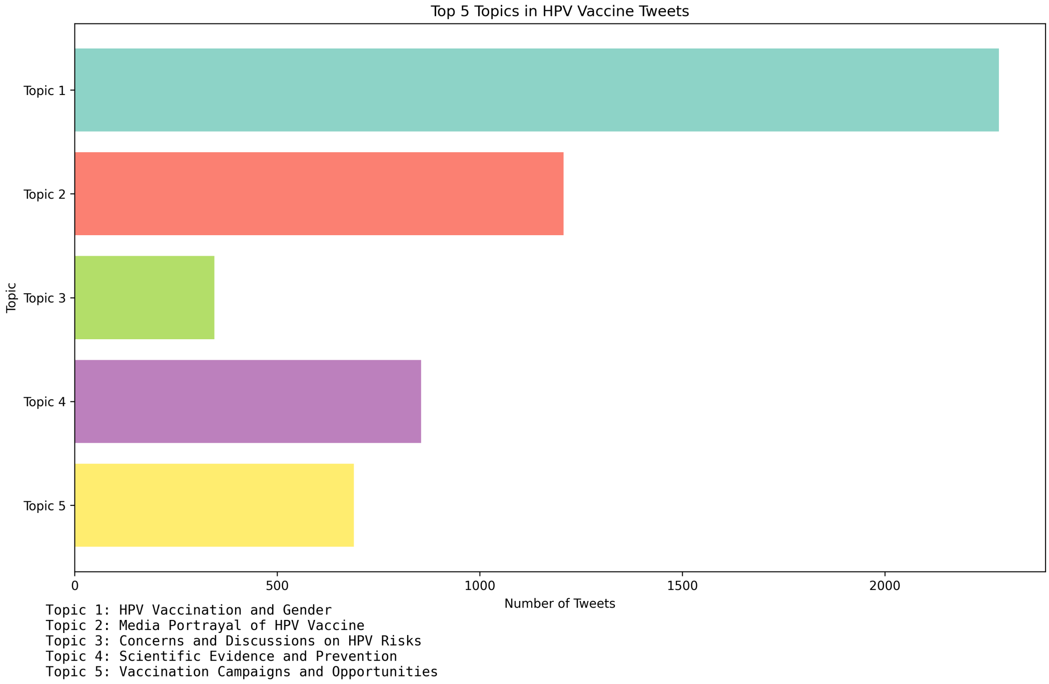

Figure 2: Top topics in HPV vaccine discussions

4. Chat Analysis

Frequent Questions and Topics

User inquiries about HPV predominantly focus on understanding the clinical presentation, prevention, and management of the infection across different age groups and populations. The most frequent topic, accounting for 20% of questions, concerns HPV symptoms and awareness. Users commonly ask about the typical signs of HPV infection, symptom variations by age, and manifestations related to persistent high-risk types such as HPV16. For example, healthcare workers and older adults frequently seek guidance on what symptoms to monitor, reflecting a need for clarity on the often asymptomatic nature of HPV.

Closely following, at 18%, are questions about HPV vaccine effectiveness and age-specific recommendations. Users express interest in how well vaccines protect against both low-risk types causing warts and high-risk oncogenic types, and whether vaccination remains beneficial beyond the adolescent years. Queries such as the benefit of vaccination for individuals aged 56-65 highlight uncertainty about vaccine utility in older adults. Similarly, 18% of questions address HPV screening guidelines, including recommended screening intervals and modalities for different age brackets, underscoring users’ desire for actionable preventive care information.

Other notable topics include vaccine safety and side effects (12%), infection risks and persistence (12%), and vaccination guidelines tailored for adults and healthcare workers (10%). General questions about HPV’s natural history and long-term risks constitute 10%

of inquiries, indicating ongoing concerns about asymptomatic infections and theirpotential consequences. Collectively, these patterns reveal that users seek comprehensive,age- and occupation-specific information on HPV symptoms, prevention strategies,vaccine efficacy, and screening protocols.

User Worries and Concerns

Users' primary concerns cluster around vaccine safety, side effects, and efficacy, with varying degrees of severity. Medium-severity worries focus on safety and side effect profiles, particularly for adults and healthcare workers receiving the vaccine later in life. Questions such as "Are there any specific considerations or precautions for healthcare workers receiving the HPV vaccine later in adulthood?" and "What are the potential side effects for someone in my age group?" illustrate apprehension about adverse reactions and immune response differences by age.

High-severity concerns predominantly relate to doubts about vaccine efficacy in older adults and the practical benefits of vaccination beyond the typical adolescent window. Users frequently ask, "At my age, is it still beneficial to get the HPV vaccine?" and "What kind of protection can I realistically expect if vaccinated now?" These questions reflect uncertainty about the value of vaccination after potential prior HPV exposure and the degree of cancer risk reduction achievable in later adulthood.

Additional high-severity worries encompass general HPV-related issues such as symptom recognition, screening frequency, and risk assessment, especially among healthcare workers and older adults. For instance, users inquire about appropriate screening intervals given the asymptomatic nature of most infections and seek guidance on monitoring strategies to detect HPV-related complications early. Concerns about men's risk of persistent high-risk HPV infections and the availability of screening tests also emerge, highlighting gaps in awareness about HPV's impact across genders.

Common Misunderstandings

Several prevalent misconceptions were identified that may hinder effective HPV prevention and management. A highly frequent misunderstanding is the belief that HPV infections typically cause noticeable symptoms. In reality, most HPV infections are asymptomatic and resolve spontaneously; high-risk infections often remain silent until advanced disease develops. This misconception leads users to underestimate the importance of regular screening, as evidenced by repeated questions about symptom monitoring.

Another widespread error is the assumption that HPV vaccination can treat or clear existing infections. Users often ask whether vaccines are effective after prior exposure or in the presence of persistent infection. The correct information is that HPV vaccines are prophylactic, preventing new infections but not clearing established ones. This misunderstanding may contribute to unrealistic expectations regarding vaccination benefits in older adults or those with prior HPV exposure.

There is also confusion about vaccination recommendations for older adults, with some users believing routine vaccination is beneficial beyond age 45. Current guidelines recommend routine vaccination primarily up to age 26, with shared decision-making for adults aged 27-45, and generally do not endorse vaccination beyond 45 due to limited benefit. Clarifying this can help align user expectations with evidence-based practices.

Concerns about vaccine safety are sometimes amplified by misconceptions that HPV vaccination causes persistent or severe long-term side effects. The evidence supports an excellent safety profile with mostly mild, transient reactions. Addressing these fears with clear, evidence-based communication is essential to improve vaccine acceptance.

Additional misunderstandings include underestimating men’s susceptibility to HPV and related cancers, and overestimating the availability and utility of routine HPV screening for men. Users also sometimes believe that vaccination obviates the need for continued cervical cancer screening, which is inaccurate since vaccines do not protect against all oncogenic HPV types. Finally, some users assume all HPV infections clear quickly without need for follow-up, overlooking the risk posed by persistent high-risk infections.

These misconceptions highlight critical areas for targeted education, emphasizing the asymptomatic nature of HPV, the preventive (not therapeutic) role of vaccination, age-appropriate vaccine recommendations, and the continued importance of screening regardless of vaccination status. Clear, tailored communication addressing these points can enhance user understanding and promote informed health decisions.

Summary

Recent analyses across multiple data sources underscore the critical importance of advancing human papillomavirus (HPV) vaccination efforts to reduce the global burden of HPV-associated cancers, particularly cervical cancer. While technological progress, such as the introduction of the 9-valent HPV vaccine, offers enhanced protection against a broader spectrum of oncogenic HPV types, significant challenges persist in achieving optimal vaccine coverage. Notably, the marked decline in vaccination rates observed in countries like Japan portends a potential rise in preventable cervical cancer morbidity and mortality, emphasizing the urgent need for coordinated public health interventions. These findings highlight a dual imperative for medical institutions and policymakers: to both leverage improved vaccine formulations and to implement effective strategies that address vaccine hesitancy and accessibility barriers.

Research advances reveal that multifaceted approaches are essential to improving vaccine uptake and expanding the clinical utility of HPV vaccines. Targeted outreach to underserved and high-risk populations, including LGBTQ communities, and culturally sensitive education tailored to diverse healthcare settings are pivotal. Digital health innovations, such as mobile applications designed to enhance parental knowledge and intention to vaccinate, demonstrate promise in overcoming behavioral obstacles. Furthermore, emerging evidence of therapeutic benefits of HPV vaccines in treating recalcitrant warts suggests potential expansion of clinical indications beyond prevention. Concurrently, refined immunological assays enable more precise monitoring of vaccine-induced immunity, supporting evidence-based evaluation of vaccination programs. Economic analyses advocate for context-specific cost-effectiveness assessments to guide resource allocation, particularly in low- and middle-income countries where financial constraints challenge program scale-up.

Social media and public engagement data reveal predominantly positive sentiment toward HPV vaccination, with ongoing discourse emphasizing gender-inclusive vaccination strategies and the influential role of media in shaping public perceptions. However, the persistence of misinformation—primarily concerning vaccine safety and efficacy—remains a significant obstacle, necessitating proactive, evidence-based communication from trusted healthcare providers. Analysis of user inquiries further identifies critical knowledge gaps and misconceptions, including misunderstandings about the asymptomatic nature of HPV infections, the prophylactic (not therapeutic) role of vaccines, age-appropriate vaccination guidelines, and the continued necessity of screening post-vaccination. These insights underscore the need for clear, targeted educational initiatives that address specific concerns of diverse populations, including healthcare workers and older adults.

For medical professionals and policymakers, these findings translate into actionable recommendations: prioritize the integration of the 9-valent HPV vaccine into national immunization schedules; develop culturally competent, population-specific education and outreach programs; harness digital tools to enhance vaccine literacy and acceptance; and establish robust surveillance systems to monitor vaccine coverage, efficacy, and

misinformation trends. Additionally, expanding training for healthcare providers toeffectively communicate vaccine benefits and address hesitancy is essential. Policymakersshould also consider flexible, locally tailored economic models to optimize investment inHPV vaccination programs, ensuring sustainability and equity. Collectively, these strategieswill be instrumental in reversing declining vaccination trends, maximizing the public healthimpact of HPV vaccines, and ultimately reducing the incidence of HPV-related cancersworldwide.
