# Supplementary figures and images for: Japanese-Language AI Agent System for Human Papillomavirus Vaccine Infoveillance and Public Communication: Development and Feasibility Evaluation
Source: JMIR Infodemiology. 2026 May 21;6:e90295. doi: 10.2196/90295 (PMC13193703; doi:10.2196/90295)

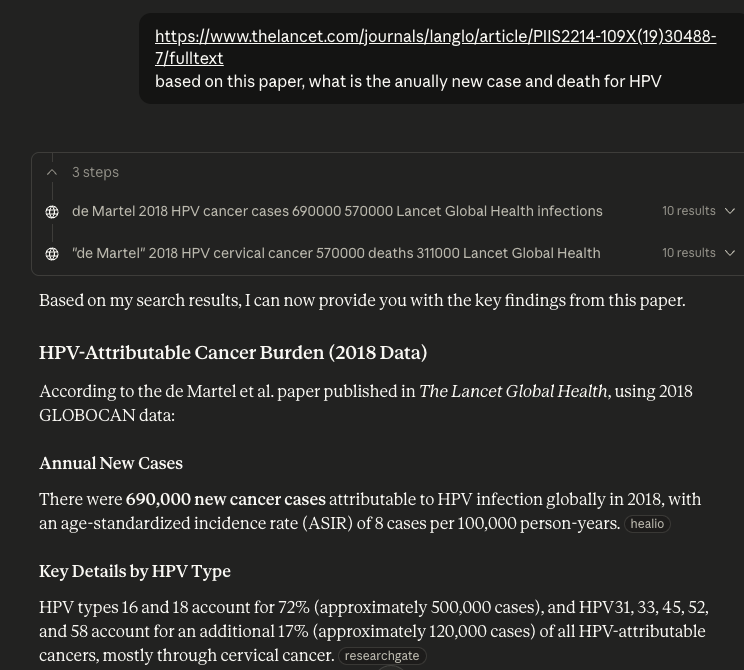


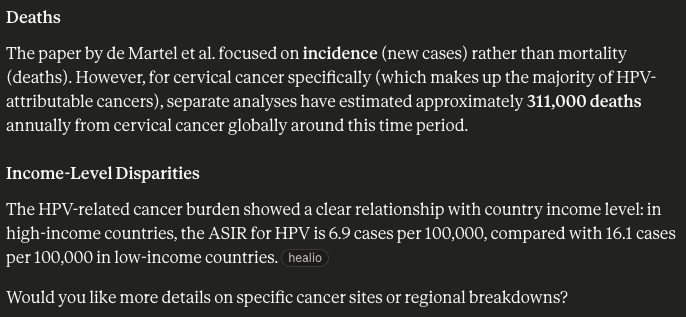


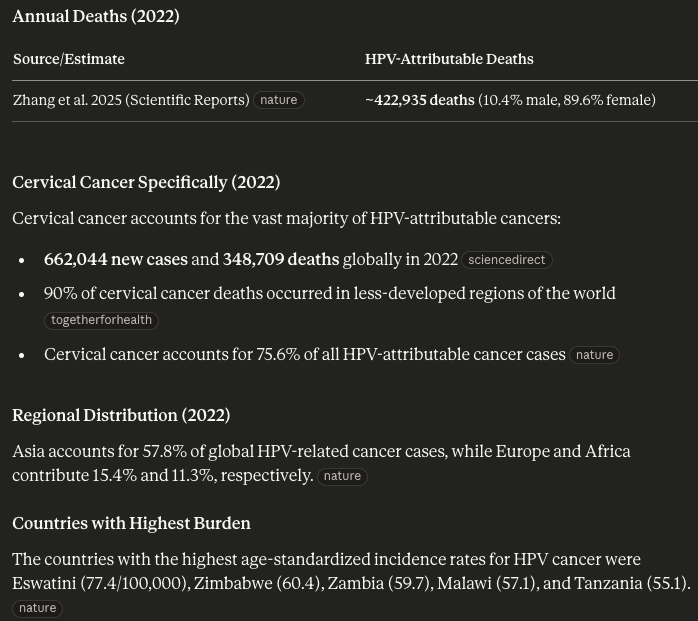

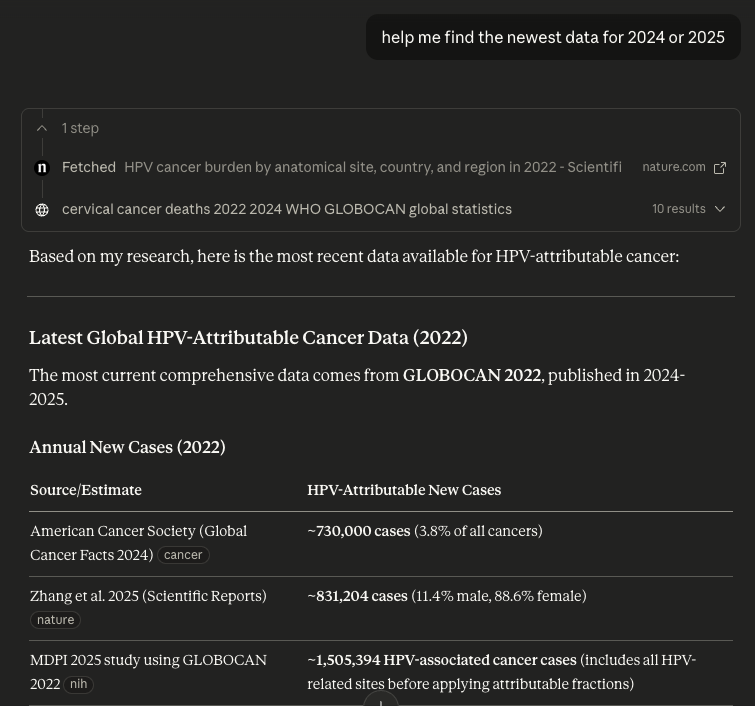


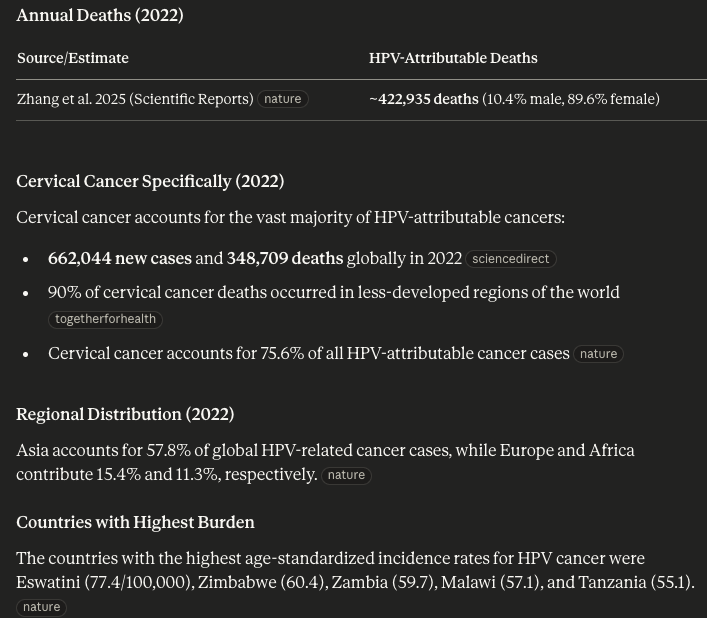


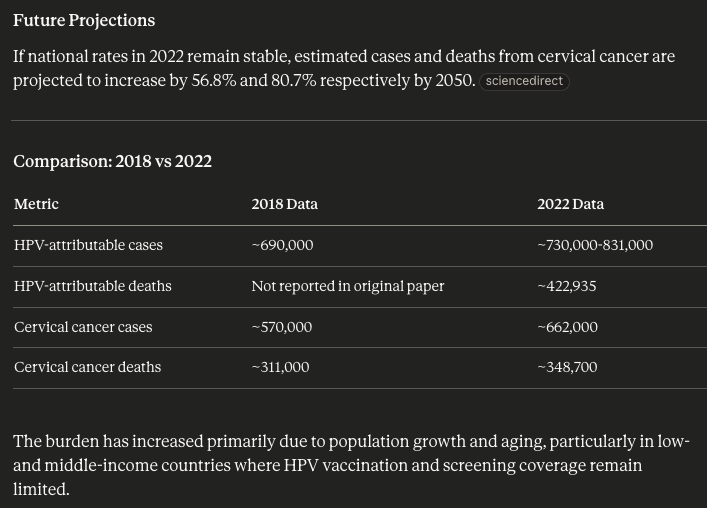


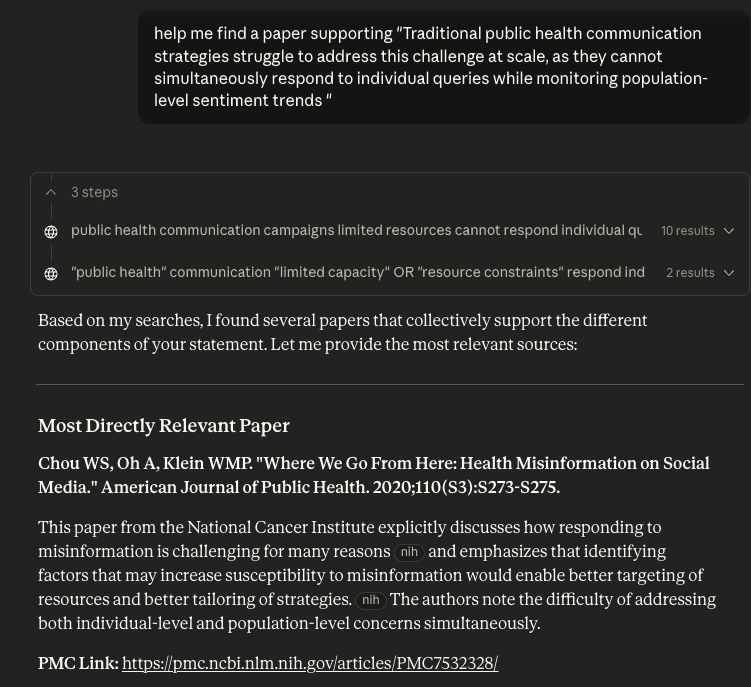


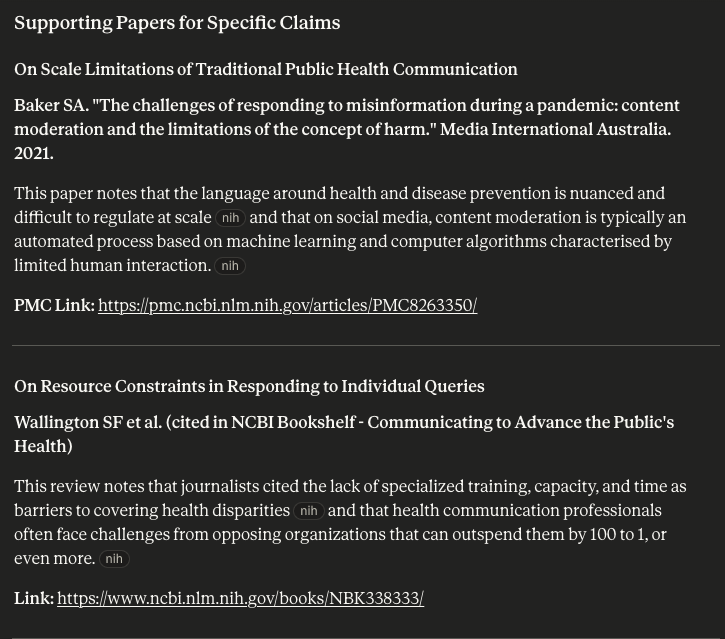


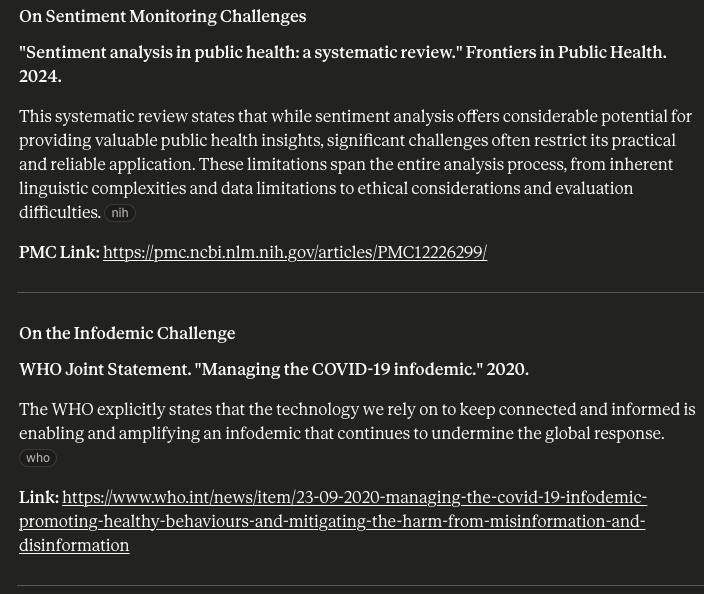


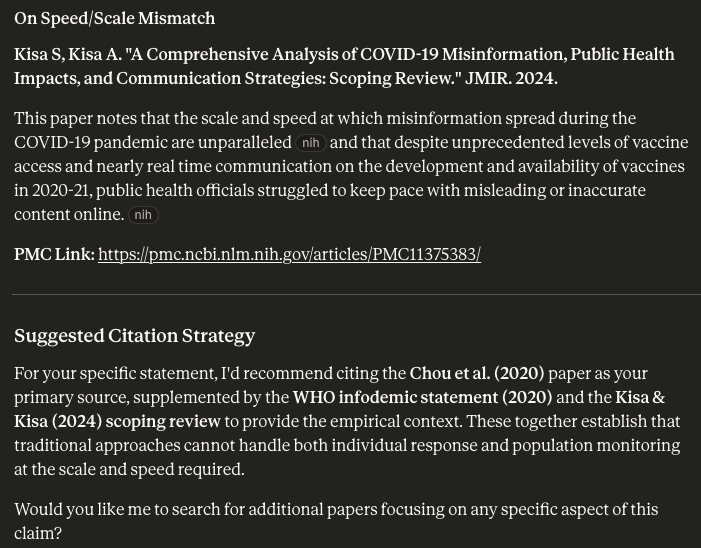


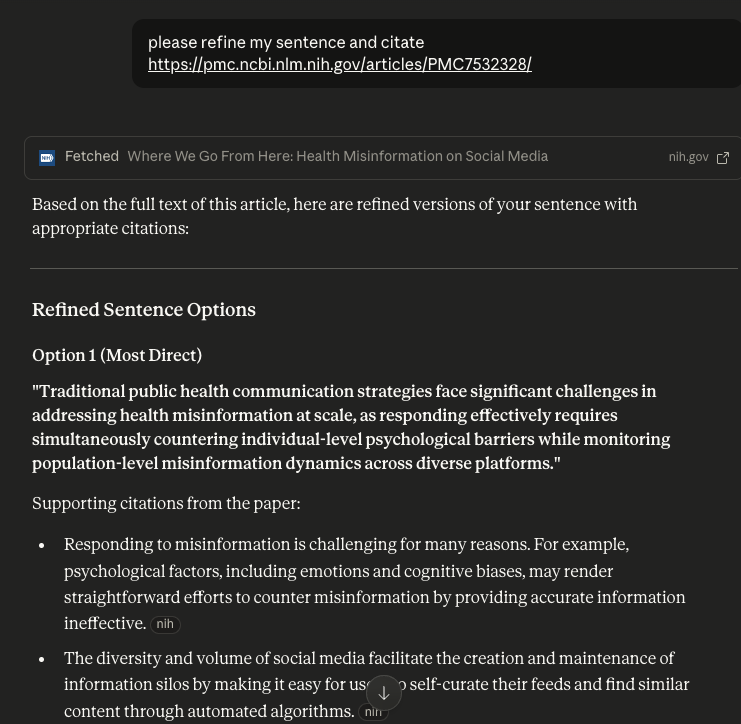


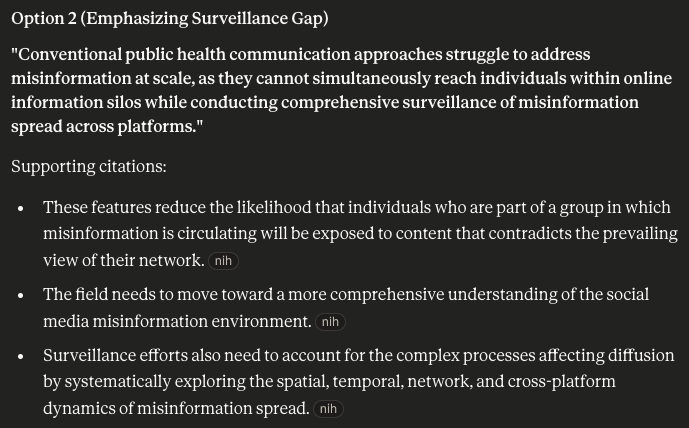


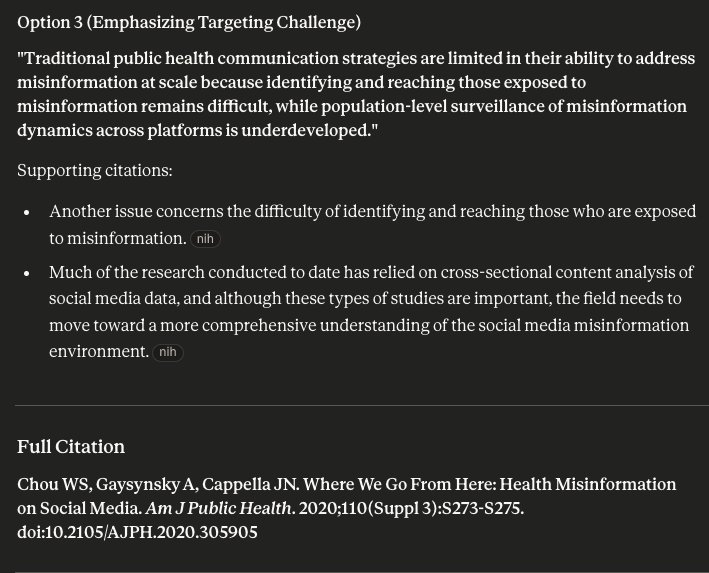


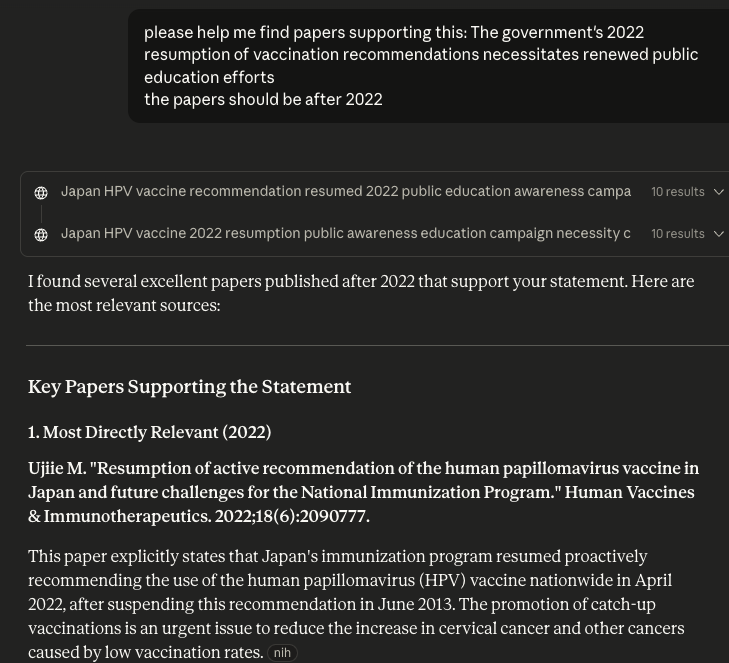


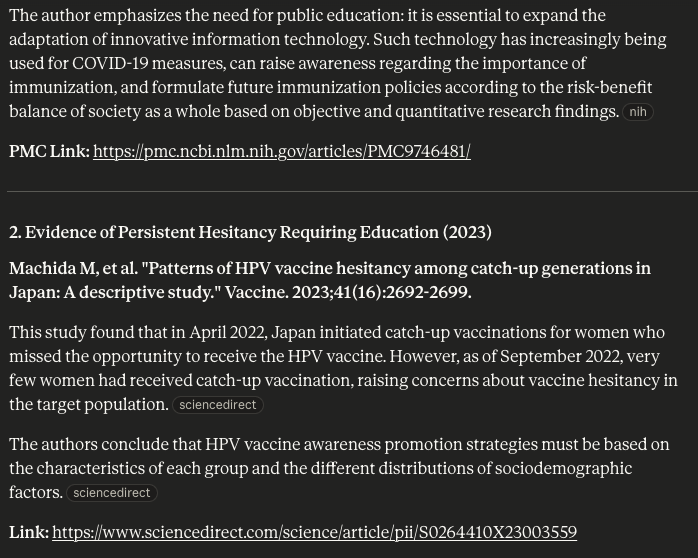


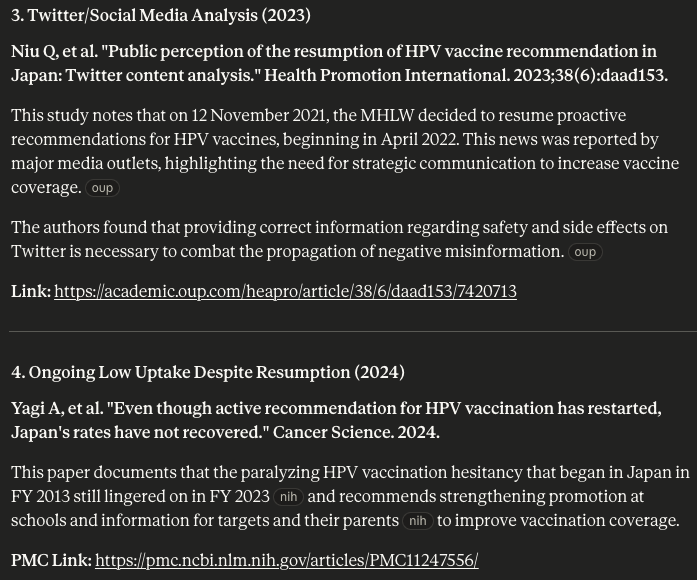


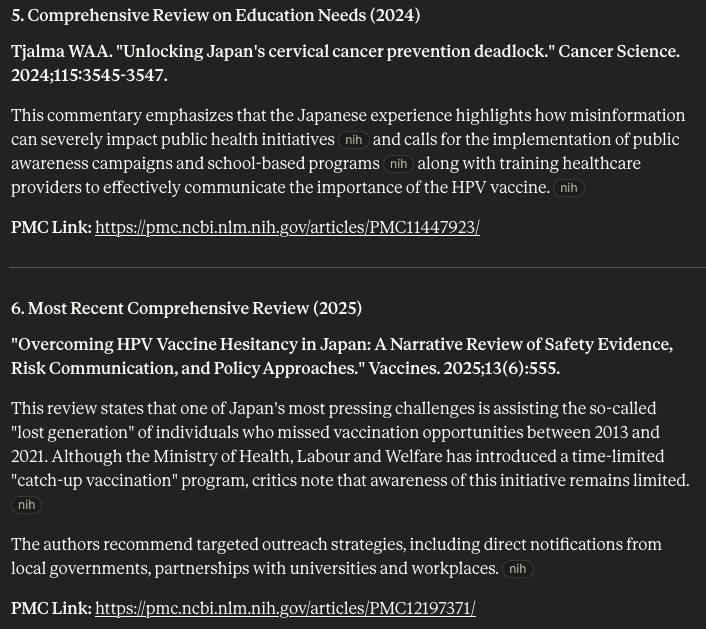


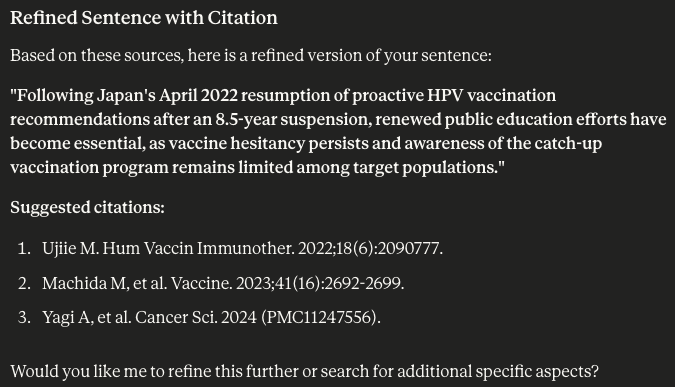

Supplement: Multimedia Appendix 11 [file infodemiology-v6-e90295-s011.docx]
